# Rheumatism

**Published:** 1856-06

**Authors:** Edwin R. Maxson

**Affiliations:** Geneva, N. Y.


### TREATMENT OF RHEUMATISM.

BY EDWIN R. MAXSON, M. D., OF GENEVA, N. Y.

Having furnished, for the April No. of the *Lancet*, my views of the pathology of rheumatism, I here offer the plan of treatment which I adopted several years since, founded mainly on those views.

And, though a short sketch from me was published on this subject, in the March No. of the Buffalo Medical Journal of 1853, it was not sufficiently definite to answer my mind, after three years, further observation of this disease, and its various complications.

Besides, I only spoke of the treatment of acute rheumatism, without referring to its various complications, or its sub-acute and chronic varieties.

I may here state, however, that the treatment which I then suggested, for acute rhematism, occurring from atmospheric vicissitudes, has fully answered my expectations, and helped to confirm me in my views, of the pathology of rheumatism as stated in the April No. of the *Lancet.*

According to my view, the immediate cause of the rheumatic inflammation, in all cases; is congestion, irritation or inflammation at some point in the brain or spinal marrow, or their fibrous envelope; together with the morbid agent or agents, which operate to produce this condition of the cerebro-spinal system, whether it be retained perspirable matter, a miasmatic agent, or any other morbid influence; but that the irritation in the nerves, or neuralgic pains, which, according to my observations, is invariably set up by this local cephalic or spinal irritation, is the principal immediate cause of the rheumatic inflammation, and always dictates its location, and very much its character.

I therefore treat all cases of rheumatism on the following principles:

If the rheumatism be acute, and the patient of a strong constitution, I take one or two ounces of blood by cups, from near the spine, at the point supplying nerves to the affected part, for the purpose of removing the local congestion, irritation, or inflammation of the spine or brain, and thereby checking the neuralgic irritation, and consequent rheumatic inflammation which is being developed.

If, however, the patient be one of a feeble constitution, dry cups only should be applied at the same point, and for the same purpose; the application being repeated if necessary, in twelve or twenty-four hours.

In many cases, this, if applied early, will arrest the disease, if the general cause which has operated to produce it has been slight, and there is considerable hereditary or accidental local cerebral or spinal irritation.

And, in those cases, in which retained perspirable matter, a miasmatic agent, or any other general cause is operating, it will generally check the development or progress of the local disease, while the general morbid condition or agent, is being corrected, removed or counteracted.

The next step then, is to remove or counteract the general morbid agent or influence, and for this purpose, a cathartic of Sulphate of Magnesia will generally do well; but if there is a bilious condition, it should be preceded by two or three Blue pills, or a full dose of calomel, and its operation followed by the sulphate of quinine, and Dover's powders, of each, gr. v. every six hours, to counteract the miasmatic influence, equalize the circulation and promote perspiration.

To aid in promoting perspiration and also the urinary secretion, the nitrate of potash in vi. gr. doses may be given every six hours, alternating with the quinine and Dover's. I generally give it in a teacup of crust coffee, or warm gruel, which serves the patient and as food and drink, during the acute inflammatory stage.

The quinine, of course, should be omitted, except in bilious, typhoid, and very debilitated cases; and in cases not requiring the quinine thirty drops of the wine of colchicum may be given every six hours, with the nitrate of potash.

Having thus subdued the acute stage of the disease, and in most sub-acute and chronic cases, the iodide of postassium in gr. x. doses every six hours, in place of the nitrate with the wine of colchicum, gtt. xx will do well, by stimulating the absorbent lymphatic and glandular system, to remove any morbid thickening that may exist.

In all decidedly chronic cases of rheumatism, of a general or local character, after dry cupping, or blistering the spine if indicated, I have generally succeeded with the iodide of potassium, in gr. v doses, three times per day, given in the compound decoction of sarsaparilla.—*Ib*

*Geneva*, Feb. 27, 1856.

